# Proteomic and bioinformatic analysis of epithelial tight junction reveals an unexpected cluster of synaptic molecules

**DOI:** 10.1186/1745-6150-1-37

**Published:** 2006-12-08

**Authors:** Vivian W Tang

**Affiliations:** 1 Department of Cell Biology, Harvard Medical School, Boston, MA 02115, USA

## Abstract

**Background:**

Zonula occludens, also known as the tight junction, is a specialized cell-cell interaction characterized by membrane "kisses" between epithelial cells. A cytoplasmic plaque of ~100 nm corresponding to a meshwork of densely packed proteins underlies the tight junction membrane domain. Due to its enormous size and difficulties in obtaining a biochemically pure fraction, the molecular composition of the tight junction remains largely unknown.

**Results:**

A novel biochemical purification protocol has been developed to isolate tight junction protein complexes from cultured human epithelial cells. After identification of proteins by mass spectroscopy and fingerprint analysis, candidate proteins are scored and assessed individually. A simple algorithm has been devised to incorporate transmembrane domains and protein modification sites for scoring membrane proteins. Using this new scoring system, a total of 912 proteins have been identified. These 912 hits are analyzed using a bioinformatics approach to bin the hits in 4 categories: configuration, molecular function, cellular function, and specialized process. Prominent clusters of proteins related to the cytoskeleton, cell adhesion, and vesicular traffic have been identified. Weaker clusters of proteins associated with cell growth, cell migration, translation, and transcription are also found. However, the strongest clusters belong to synaptic proteins and signaling molecules. Localization studies of key components of synaptic transmission have confirmed the presence of both presynaptic and postsynaptic proteins at the tight junction domain. To correlate proteomics data with structure, the tight junction has been examined using electron microscopy. This has revealed many novel structures including end-on cytoskeletal attachments, vesicles fusing/budding at the tight junction membrane domain, secreted substances encased between the tight junction kisses, endocytosis of tight junction double membranes, satellite Golgi apparatus and associated vesicular structures. A working model of the tight junction consisting of multiple functions and sub-domains has been generated using the proteomics and structural data.

**Conclusion:**

This study provides an unbiased proteomics and bioinformatics approach to elucidate novel functions of the tight junction. The approach has revealed an unexpected cluster associating with synaptic function. This surprising finding suggests that the tight junction may be a novel epithelial synapse for cell-cell communication.

**Reviewers:**

This article was reviewed by Gáspár Jékely, Etienne Joly and Neil Smalheiser.

## Open peer review

Reviewed by Gáspár Jékely, Etienne Joly and Neil Smalheiser. For the full reviews, please go to the Reviewers' comments section.

## Background

**Figure 1:**
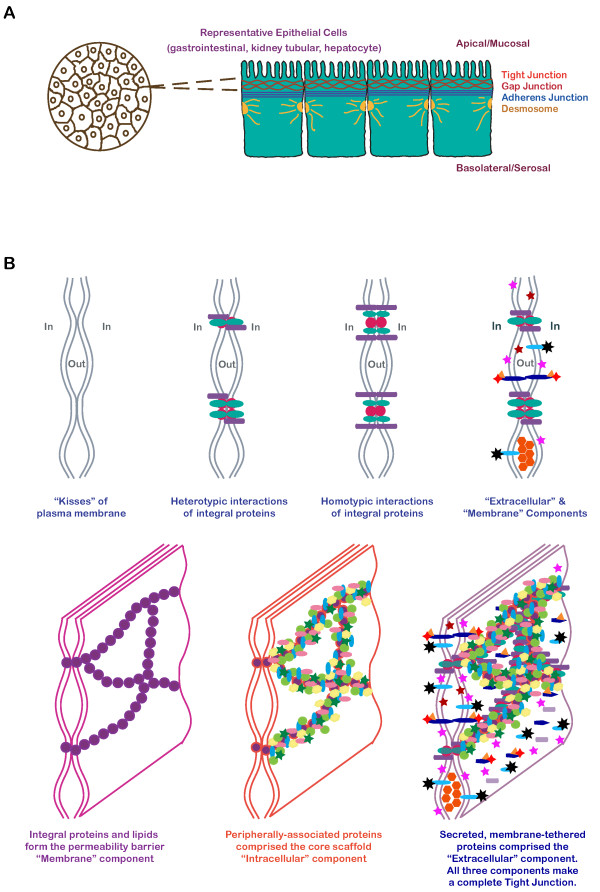
**Structure of the tight junction**. (A) Epithelial cells when grown to confluent density form polarized intercellular junctions with tight junctions and gap junctions at the apical-most lateral position, followed by adherens junctions and desmosomes. (B) The tight junction consists of three major components: extracellular, membrane, and intracellular components. Integral membrane proteins and lipids form the membrane component. Secreted and membrane tethered extracellular proteins form the extracellular component. Cytoplasmic scaffolds and associated proteins form the intracellular component.

The tight junction is a specialized cell-cell interaction that is found in almost all types of epithelial cells [1]. An electron dense plaque of ~100 nm [2] underlies the cytoplasmic domain of the tight junction. This sub-membrane structure can be seen as a meshwork of densely packed proteins in detergent-extracted cells [3]. Small vesicular structures and pinocytosis have been seen to associate with the tight junction, suggesting that it may be a region of selective intercellular exchange [4,5]. The membrane domain of the tight junction is subdivided into discreet corralled sub-domains, which are physically demarcated by polymerized membrane components. These sub-domains are sometimes packed with membrane proteins, which appear as intramembrane particles [6-10]. The complexity of the tight junction is illustrated in Figure 1.

Despite being an enormous structure, the tight junction is dynamically regulated. In normal epithelial turnover, the tight junction moves down the lateral membranes of the extruding cell while the cell moves up and out of the epithelium [11,12]. During cell division, new tight junctions are formed between the daughter cells and their neighbours before cytokinesis is completed [13]. Tight junctions can readily be opened during leukocyte transmigration and reseal quickly to re-establish the permeability barrier [14]. The tensile nature of the tight junction can be seen during mechanical stretching where the intramembrane strands move laterally to rearrange from a compact network to an elongated network [15]. During wound healing, tight junction proteins are rapidly re-localized to a purse string structure that eventually closes the wound [16,17]. Dynamic formation of the tight junction is observed in embryonic development where its proteins re-position from a basal location to an apical location [18]. Assembly of the tight junction is a complex process which is influenced by multiple factors including vesicular trafficking [19] and extracellular proteases [20,21].

Beside the classical barrier function [22-27], the tight junction is emerging as a regulator of cell growth and differentiation [28]. Tight junction proteins ZONAB [29,30], cingulin [31], and claudin-11/OSP [32], have been shown to regulate cell proliferation. ZO-1 [33,34] and TGFbeta type II receptor [35] are involved in epithelial-mesenchymal transition. Claudin-1 has been shown to regulate transformation and metastasis of colon cancer cells [36]. Occludin [37] and Claudin-6 [38] are involved in differentiation of the gastric epithelium and epidermis, respectively. Nevertheless, there are many processes associated with the tight junction that have little or no molecular and mechanistic explanations. These are (i) intercellular mechanisms that allow cell-cell communication, (ii) signaling pathways that lead to regulation of contact inhibition of cell growth and cell migration, (iii) molecular events that lead to assembly of tight junction scaffold, (iv) molecular events that lead to generation of tight junction membrane microdomain, intramembraneous strands, permeability barrier, and paracellular channels, (v) extracellular adhesive interactions and their regulation, (vi) intracellular cytoskeletal interactions and their regulation, (vii) contribution to morphogenesis and tensile strength of the epithelial sheet, (viii) maintenance of a steady-state through regulation of protein synthesis and membrane recycling, and (ix) generation of polarity during differentiation and epithelialization. The current knowledge of known tight junction proteins is far from explaining even one of these complex processes.

Although progress has been made in identifying tight junction proteins, the current count is about 50 proteins [39], a number that is way below what is expected for a complex macromolecular structure. Of these known tight junction proteins, 21 belong to the claudin family [40] and 12 belong to the PDZ domain-containing family. Since different epithelial cells express different combinations of the ~50 proteins, the actual number of known tight junction protein in any given epithelial cell is much lower than 50. A list of known tight junction proteins and a general overview of the tight junction can be found on the Tight Junction website [41].

The lack of a comprehensive molecular characterization poses a major obstacle in the understanding of the functions and processes that are associated with the tight junction. The reason for this deficit is the difficulty with biochemical purification. There has been only one attempt in over 20 years to purify the tight junction [42]. Using tedious ultrastructural assays, Stevenson and Goodenough have managed to obtain a detergent-resistant fraction from mouse liver that is enriched in tight junction structures. This junctional fraction contains many polypeptides but the identities of the polypeptides remain largely unknown. The junctional fraction has been used as an immunogen to generate a monoclonal antibody that has led to the discovery of the first tight junction protein, ZO-1. Subsequent identification of tight junction proteins are largely by chance [43-47], or from co-immunoprecipitation experiments [48,49].

In this study, a novel protocol has been developed to purify tight junction complexes from a pure source of cultured cells, eliminating potential contamination from other cell types. A model human intestinal epithelial cell line T84 has been chosen for several important reasons. First, the databases for human are the most complete. Second, humans express, however small, a unique set of cellular proteins not found in other animals. Third, T84 is an immortal cell line and provides an unlimited source for morphological and biochemical studies that is otherwise unethical if obtained from human donors. Fourth, T84 has been used for many years in a vast number of physiological studies and possess dynamic intercellular properties including movement of cells along the crypt-villus axis, cell extrusion at the villus, as well as leukocyte transmigration.

Also in this study, a novel method has been developed to score potential candidates obtained from mass spectroscopy and fingerprint analysis. In addition, a simple algorithm has been devised to incorporate transmembrane domains, glycosylation sites and signal peptides to increase the probability of scoring potential membrane hits. Lastly, a thorough ultrastructural description has been performed to comprehensively analyze the tight junction of T84 cells. The results are cross-correlated with bioinformatics data to generate a structure-function relationship of tight junction-associated processes.

## Results

### Biochemical purification of tight junction complexes

**Figure 2:**
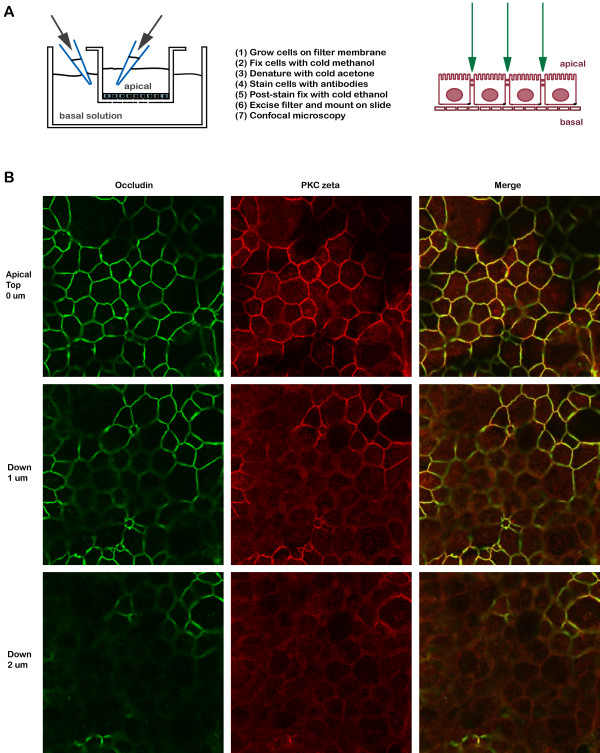
**Immunofluorescence staining of tight junction proteins**. (A) Protocol for visualization of tight junction proteins in monolayer of cells grown on filter support (see Methods for details). (B) Confocal images of T84 cells scanned at 1 μm intervals. Occludin (green) and PKC zeta (red) are localized to the apical tight junction domain. Merged images show both proteins appearing and disappearing together in the same confocal planes.

**Figure 3:**
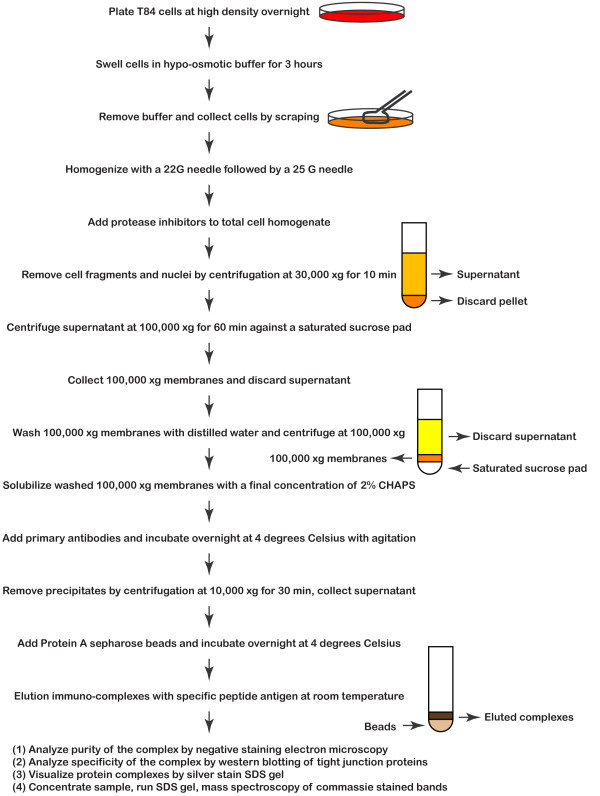
Schematic representation of the different stages of biochemical purification of tight junction complexes.

Since there is no established method to purify tight junction complexes from cultured cells, a protocol has been established empirically. First, markers for the tight junction were tested in T84 cells by immunofluorescence localization (Fig. 2A&B). Second, the antibodies were assessed in their efficiency in isolation of known tight junction components. Third, soluble proteins were excluded at an early stage of the purification to avoid identification of non-junctional complexes. After testing many cell disruption methods, buffers and detergents, separation procedures, and elution schemes, a reproducible biochemical purification protocol was established (Fig. 3, see Methods). To increase the chance of finding proteins involved in synthesis, recycling, targeting, and degradation of tight junction components, T84 cells undergoing dynamic junction assembly/disassembly were used. This was achieved by trypsinizing and re-plating confluent plates of cells at ~18 hours before utilization for biochemistry.

A hypotonic method was used to swell epithelial cells while they were still attached to the dish. This procedure helped to disrupt the rigid cytoskeletal framework of epithelial cells and increased the efficiency of cell membrane disruption. The method also allowed removal of most buffers because the cells were still attached after swelling, effectively avoiding dilution of cellular contents and proteolysis. A high pH buffer was used to facilitate breaking and resealing of membranes, thus increasing the formation of plasma membrane fragments.

After homogenization and differential centrifugation, a 100,000 × g membrane fraction enriched in tight junction components was used for the final affinity purification step using anti-PKC zeta antibodies. Anti-PKC zeta antibodies were chosen for several important technical and scientific reasons. First, PKC zeta strongly localized to the tight junction of all 4 epithelial cells tested including human intestinal T84 (Fig. 2B), C2bbe1, MDCKI, and MDCKII cells. Second, three different antibodies (from Sigma, Invitrogen, and Santa Cruz Biotech) showed similar tight junction staining. Third, PKC zeta has been shown to associate with signaling molecules such as PAR3, ASIP, and PP2A to promote formation of tight junctions [50-53], suggesting that it may interact with a variety of tight junction regulatory proteins including vesicular trafficking and assembly complexes. Fourth, the PKC zeta antibodies were anti-peptide antibodies that allowed specific elution of immunoprecipitated proteins with the peptide antigen. Fifth, known tight junction proteins including ZO-1, ZO-2, and occludin could be co-immunoprecipitated with the antibodies in the zwitterionic detergent CHAPS, a detergent of choice for isolation of large membrane protein complexes.

**Figure 4:**
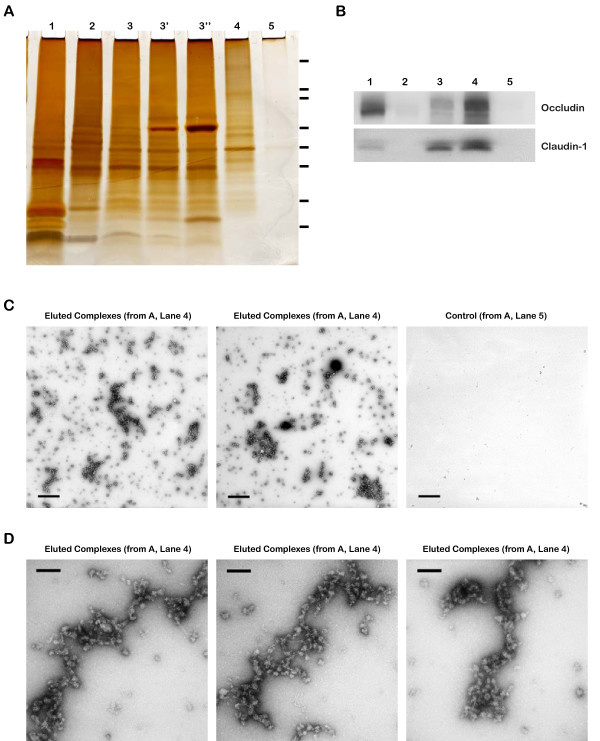
**Validation of specificity and enrichment of tight junction complexes**. (A) Silver stained gel showing steps of purification. Lane 1, whole cell lysate; lane 2, 30,000 × g supernatant; lane 3, 100,000 × g membrane; Lane 3', output 100,000 × g membrane after immuno-isolation, Lane 3", output 100,000 × g membrane after control immuno-isolation; lane 4, immuno-isolated tight junction complexes; lane 5, control immuno-isolation. MW markers are 200, 116, 96, 66, 45, 38, 25, 14. (b) Western blots of tight junction markers occludin and claudin-1. Lanes 1–5, same as A. (C) Negative staining of complexes showing assemblies of a heterogeneous population of proteins in various sizes. Control immunopurification shows relatively clean background. Scale bars, 100 nm. (D) Higher magnification of tight junction complexes shows globular proteins linked together forming beaded-necklace arrays reminiscent of tight junction seen *in vivo *(see Results).

After immuno-isolation and elution, the tight junction fraction was analyzed by (i) negative stain electron microscopy, (ii) western blotting of proteins separated by SDS-PAGE, (iii) silver staining of proteins separated by SDS-PAGE, and (iv) peptide mass spectroscopy. The purified tight junction fraction appeared specific because a parallel control purification using pre-immune antibodies showed a reasonably clean background (Fig. 4A–C). The complexes were enriched in known tight junction proteins as shown by western blots of occludin and claudin-1 (Fig. 4B). Silver stain analysis showed a very complex protein population with strong and weak bands ranging from 20 kD to greater than 300 kD (Fig. 4A &Additional file 1). Visualization of the complexes under electron microscopy showed a heterogeneous population of assemblies in various sizes (Fig. 4C & D). Higher magnification showed globular units linked together forming linear arrays. Occasionally, a few larger globular assemblies that appeared to consist of many subunits were also seen. The overall appearance of the *in vitro *structures and the dimensions of individual globular units closely resembled the *in vivo *meshwork of densely packed proteins seen by freeze-fracture technique [3].

### Identification and scoring of hits

**Figure 5:**
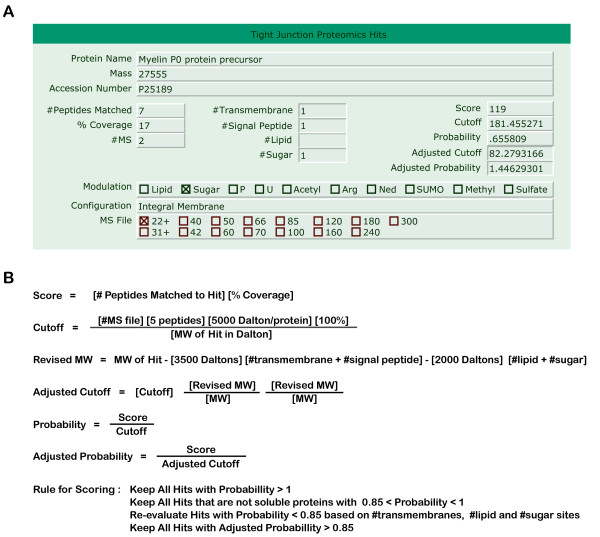
**Data collection and criteria for scoring of hits**. (A) Filemaker prototype for data entry. (B) Equations and scoring criteria for generation of the tight junction proteomics database. #Peptides matched to hit is defined as the number of peptides found using fingerprint analysis that has been matched to a particular protein in the SwissProt UniProt database. %Coverage is defined as the percent of amino acid sequence of the particular protein that has been represented by the peptides found. #MS file is defined as the number of file(s) used in fingerprint analysis, which is the same as the number of times a particular band is analyzed by mass spectroscopy (see Supplementary Figure S2A). MW of hit in Daltons is defined as the molecular mass of the matched protein. The cutoff is calculated from an average peptide mass of 1000 Daltons and a 10% coverage of a cellular protein with modal molecular mass 50 kD, i.e. 5 peptides and 5000 Daltons per protein. The "revised MW" is based on the molecular mass of the particular protein, corrected for the number of transmembrane, signal peptide, lipid modification, and sugar modification groups. A correction of 3500 Daltons is used for transmembrane and signal peptide regions based on the average number of amino acids in those sequences. A correction of 2000 Daltons is used for lipid and sugar modifications to cover two average peptide lengths when the modifications are at the ends of the peptides. Lipid and sugar modification sites are obtained from SwissProt UniProt database. The "adjusted cutoff" is calculated from the cutoff and the ratio of the "revised MW" to MW, adjusted once for the MW and a second time for the coverage.

Protein bands from three separate purifications were submitted for in-gel digestion and peptide mass spectroscopy (see Methods &Additional file 1 for details). Subsequently, fingerprint analysis was performed using Protein Prospector (UCSF) to identify candidate proteins. A novel scoring system was developed to assess the candidates individually. A probability for each candidate was calculated from a "score" and a "cutoff" which was used to determine whether the protein could be counted as a *bona fide *tight junction hit (Fig. 5A & B). The "score" for each candidate was calculated from the number of peptides matched, molecular mass of the protein, and the percent coverage. The "cutoff" for each candidate was calculated from the number of purifications used for fingerprint analysis and based on a 10% coverage of a cellular protein with modal molecular mass, 50 kD. Candidates with probability greater than 1 were counted as *bona fide *"hits". Candidates with probability between 1 and 0.85 that were not soluble proteins were also counted as *bona fide *"hits". For hits that fell below the initial scoring criteria, an "adjusted molecular mass" was used for calculation of an "adjusted score" for proteins that contain signal peptides, transmembrane domains, lipid and carbohydrate modifications (Fig. 5A & B). Using this scoring system, a total of 912 hits were identified. A complete list of hits can be found on the TJ Proteomics website [54].

### Bioinformatic analysis of hits

**Figure 6:**
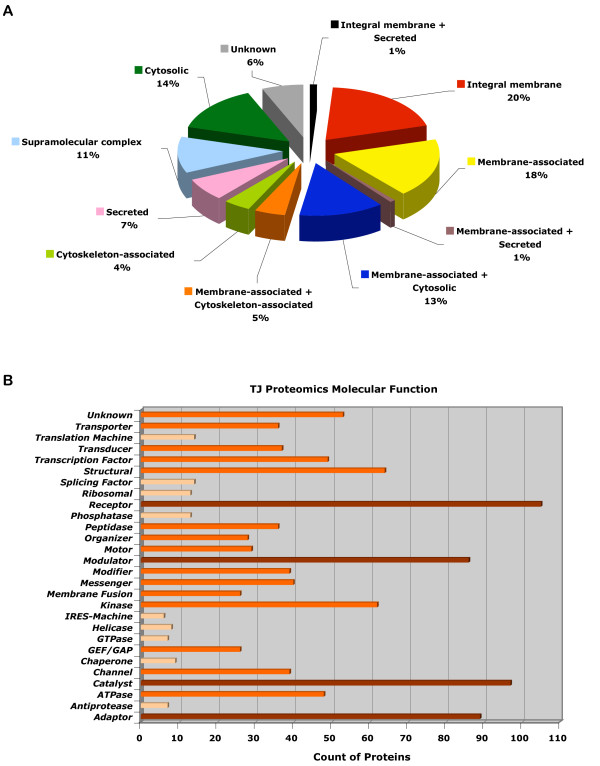
**Categorization of hits based on their configurations and molecular functions**. (A) Pie graph representing all 912 hits found in this study showing 21% integral membrane proteins, 37% membrane-associated proteins, 9% cytoskeleton-associated proteins, 8% secreted proteins. Each of the 912 hits has been counted once to generate this pie chart. (B) Bar graph representing all 912 hits found in this study showing a wide spectrum of molecular functions including prominent clusters of receptor and structural proteins, kinases, modulators and adaptors. Each protein can be counted more than once if it has functions fitting more than one category.

**Figure 7:**
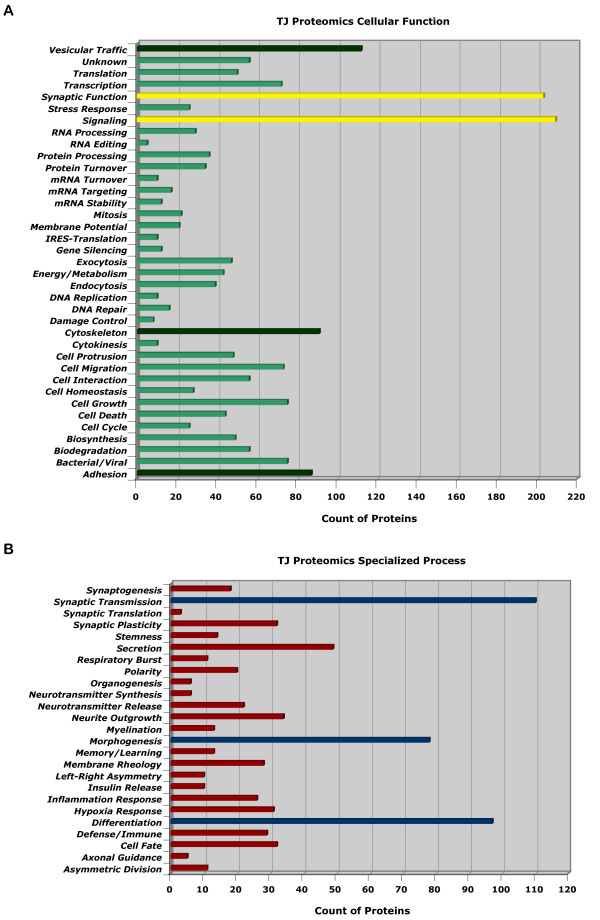
**Categorization of hits based on their cellular functions and involvements in specialized processes**. (A) Bar graph representing all 912 hits found in this study showing major clusters in signaling and synaptic functions. Prominent clusters are also found in adhesion, cytoskeleton, and vesicular trafficking categories. Each protein can be counted more than once if it has functions fitting more than one category. (B) Bar graph representing all 912 hits found in this study showing major clusters in differentiation, morphogenesis, secretion, and synaptic transmission functions. Each protein can be counted more than once if it has functions fitting more than one category.

To understand the hits from a cell biologist's point of view, bioinformatics analysis was performed. After data mining using Swiss UniProt and NCBI PubMed, each protein was binned in 4 categories: protein configuration, molecular function, cellular function, and specialized process (Fig. 6 &7, see Methods and Additional file 2 for details). As expected, the majority of hits were known integral (21%) and membrane associated proteins (37%). There were also prominent representations of cytoskeleton-associated proteins (9%), secreted proteins (7%), and proteins that form supra-molecular complexes (11%).

Analysis in the molecular function category revealed a wide distribution ranging from structural proteins, receptors, kinases, modulators, adaptors, catalysts, and transcription factors (Fig. 6B). There were also strong representations of transporters, messengers, channels, transducers, peptidases, modifiers, and motors.

**Figure 9:**
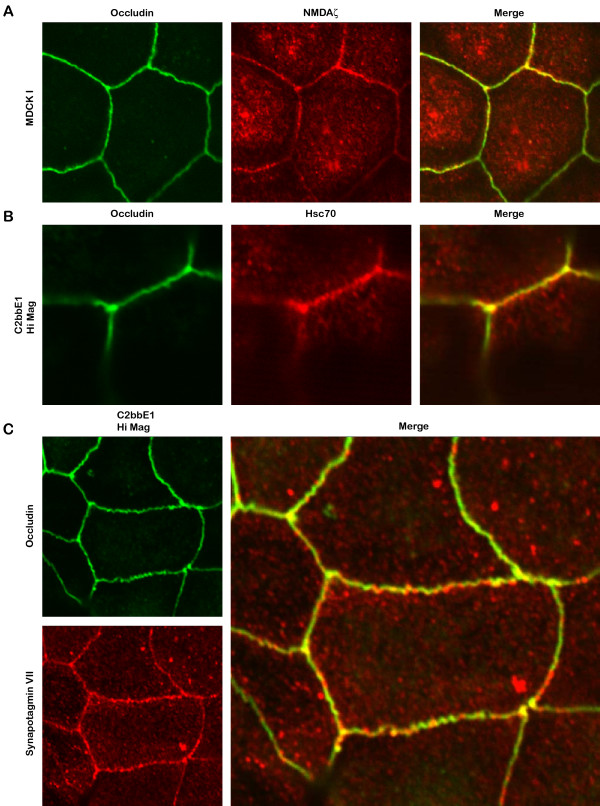
**Immunofluorescence localization of NMDA zeta receptor subunit (NR1), Hsc70, and synaptotagmin VII to epithelial tight junctions**. (A) Confocal images showing immunofluorescence localization of postsynaptic NMDA receptor zeta subunit co-localized with occludin in MDCK I cells (one week post-confluent). (B) Confocal images showing immunofluorescence localization of presynaptic Hsc70 localized to tight junction domains of C2bbE1 cells (4 weeks post-confluent). (C) Confocal images showing immunofluorescence localization of presynaptic synaptotagmin VII localized to tight junction domains of C2bbE1 cells (4 weeks post-confluent). Note the punctate patterns of NMDA zeta, Hsc70, and synaptotagmin VII.

Analysis in the cellular function category revealed major clusters in synaptic and signaling functions (Fig. 7A). Prominent clusters in vesicular traffic, cytoskeleton, and adhesion were also found. Analysis in the specialized process category revealed major clusters in synaptic transmission, morphogenesis, differentiation, and secretion (Fig. 9B).

### Confirmation of synaptic and non-synaptic hits at the tight junction

**Figure 8:**
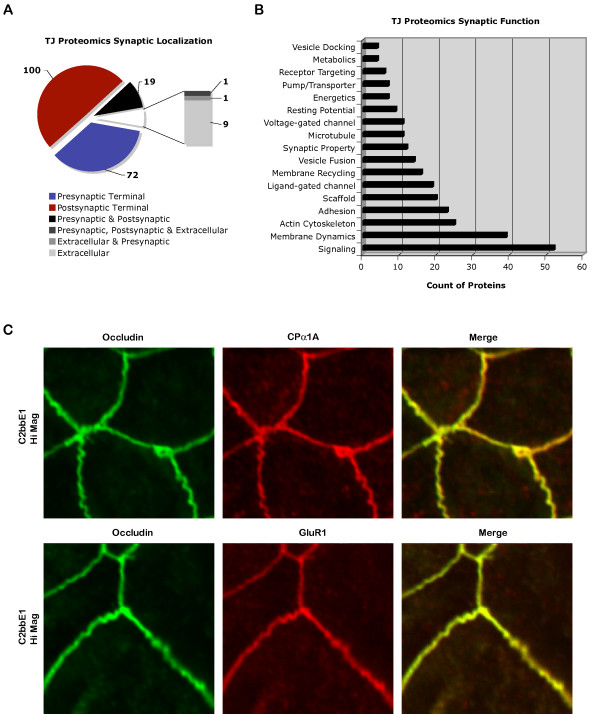
**Analysis of synaptic hits**. (A) Categorization of 202 synaptic hits based on synaptic localizations. (B) Sub-categorization of 202 synaptic hits based on synaptic functions. (C) Confocal images showing immunofluorescence localization of presynaptic P/Q-type voltage-gated calcium channel subunit CPα1A and postsynaptic AMPA receptor subunit GluR1 to tight junctions of C2bbE1 intestinal epithelial cells (4 weeks post-confluent). Tight junction formation has been monitored by measurement of transepithelial electrical resistance before the cells are utilized for staining. Occludin is used as a marker for tight junction domain. Merged yellow images show overlapping of occludin and synaptic proteins.

A more detailed analysis of the synaptic hits showed representation of both presynaptic and postsynaptic components (Fig. 8A), which covered a wide spectrum of functions for synaptic transmission (Fig. 8B).

**Figure 10:**
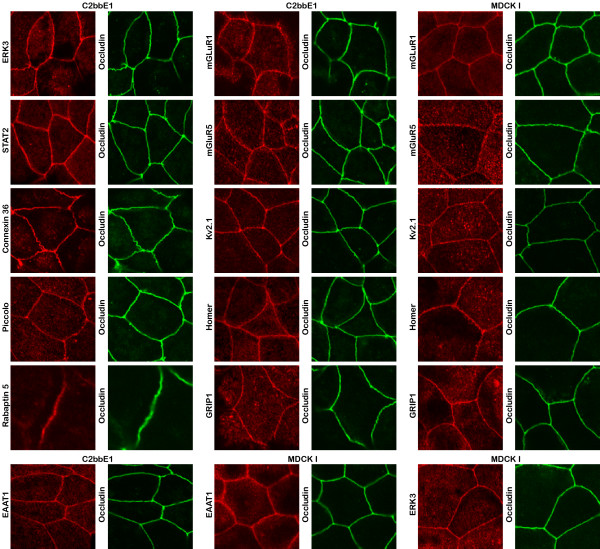
**Immunofluorescence localization of non-synaptic and synaptic hits to epithelial tight junctions**. Confocal images showing immunofluorescence localization of non-synaptic proteins (ERK3 and STAT2) as well as synaptic proteins (mGluR1, mGluR5, Kv2.1, homer, GRIP1, EAAT1, rabaptin-5, piccolo, and connexin 36) in C2bbE1 (4 weeks post-confluent) and MDCK I (one week post-confluent) cells.

Hits in several of the major clusters were confirmed by immunofluorescence in 4 different cells (T84 human intestinal, C2bbe1 human intestinal, MDCK I and MDCK II canine kidney). Each hit was stained at least twice in 4 different cells at 2 different time points (1 day and >7 days post-confluent). Tight junction formation in each case was monitored by measurement of transepithelial electrical resistance before the cells were utilized for staining. Representative immunofluorescence stainings of some of the hits showed overlapping localization with the tight junction marker occludin (Fig. 8C, 9, &10). Both presynaptic and postsynaptic proteins were present at the tight junction domain (Fig. 8C, 9, &10). The presynaptic components included the P/Q-type calcium channel subunit CPα1A (Fig. 8C), belonging to a major voltage-gated calcium channel for neurotransmitter release in the brain [55]. There were proteins involved in docking, fusion, and recycling of synaptic membranes [56-58] including Hsc 70 (Fig. 9), synaptotagmin VII (Fig. 9), and rabaptin-5 (Fig. 10). A neurotransmitter transporter for re-uptake of glutamate EAAT1 [59] was also found to localized strongly at the tight junction (Fig. 10). The postsynaptic components included channels responsible for initiation of membrane depolarization and calcium influx [60] such as AMPA glutamate receptor subunit GluR1 (Fig. 8C) and the NMDA glutamate receptor zeta subunit (Fig. 9). There were also metabotropic glutamate receptors, mGluR1 & mGluR5 (Fig. 10), for downstream signaling to phospholipase C [61]. In addition, the voltage-gated potassium channel Kv2.1 (Fig. 10), a regulator of membrane potential and neuronal excitability, was also present [62]. Scaffold proteins including presynaptic piccolo as well as postsynaptic GRIP and Homer [63-65] were also found at the tight junction domain (Fig. 10).

Examination of temporal and spatial localization of these synaptic proteins indicated that they were differentially regulated in an epithelial sheet (unpublished observations). For example, NMDA receptor zeta subunit was targeted early during junction formation but was found only in a fraction of cells at a later stage. On the other hand, GluR1 was targeted to the tight junction later than NMDA receptors but presented homogeneously in every cell. The expression of EAAT1 was early and homogeneous. On the other hand, many proteins showed a punctate distribution, including Hsc70, EAAT1, synaptotagmin VII, mGluR1 & mGluR5. Rabaptin-5 had a punctate distribution and only presented in a small fraction of the cells. Therefore, it is likely that different cells in the epithelium express only a subset of the synaptic proteins depending on their growth and differentiation states, their localization with respect to each other, as well as the cellular activities of their neighbouring cells.

Immunofluorescence localizations of several non-synaptic proteins belonging to signaling function category also showed co-localization with occludin (Fig. 10). These included ERK3, a regulator of the cell-cycle [66,67] and STAT2, a signaling and transcription regulator [68,69].

### Enrichment of hits with occludin in heavy plasma membranes

In addition to localization studies, some of the hits were also confirmed by biochemical co-enrichment with tight junction proteins. Since quantitative measurement does not tolerate usage of detergents, which may disrupt weak and transient interactions, a membrane fractionation protocol has been devised to prepare tight junction-containing membranes in the absence of detergent (see Methods). Using confluent T84 cells as starting materials, a preparation containing heavy plasma membrane sheets enriched in occludin was used to assess co-enrichment of several hits including ERK3, STAT2, homer, mGluR5, and connexin 36 [see Additional file 3]. Some of the hits, such as hsc70 and EAAT1, were not co-enriched with occludin but were present in the heavy plasma membrane fraction. A list of all the antibodies used for immunofluorescence and western blot studies is provided [see Additional file 3].

### Identification of ultra-structures within the tight junction domain

Although T84 cells provide an excellent source for biochemical purification, the ultrastructures of their tight junctions have not been comprehensively studied. Since different epithelial cells have distinct tight junction characteristics, it is imperative to obtain a detailed description of T84 tight junctions for comparison with biochemical results. The condition for growing T84 cells on porous filters has been standardized in this study to allow enough time for: (i) epithelial proliferation and differentiation into columnar cells with polarized morphologies including apical microvilli and basally located nuclei, and (ii) formation of functional tight junction barrier as assessed by transepithelial electrical resistance. Also in this study, a protocol has been optimized for electron microscopic examination of cells that have been grown on porous supports (see Methods).

**Figure 11:**
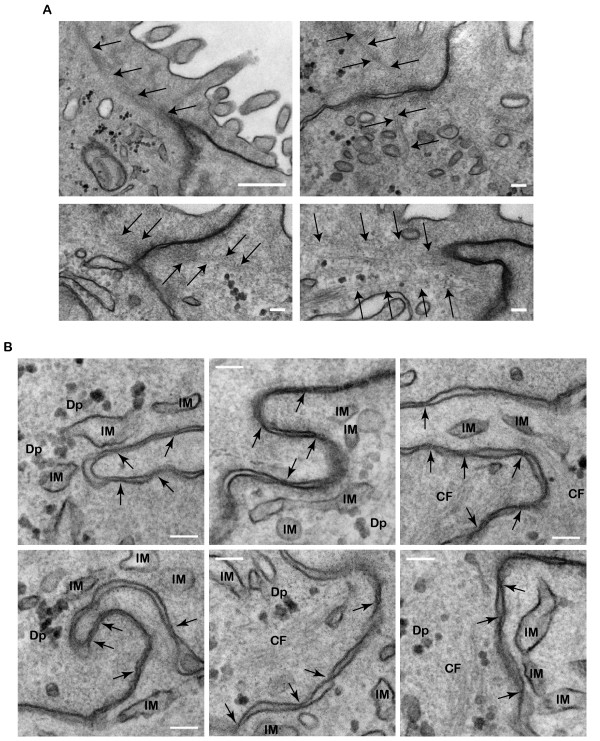
**Ultra-structural study of the tight junction**. (A) Filamentous cytoskeletal bundles (arrows) ending head on at the cytoplasmic domain of the tight junction. Arrows mark the characteristic tight junction membrane kisses. Scale bars, 100 nm. (B) Intracellular membranes, IM, of various shapes and sizes are located at the proximity of tight junction. Some intracellular membranes are located very close to the plasma membrane and appeared docked by dense materials to the tight junction. Cytoskeletal filament bundles, CF, and numerous dense particles, Dp, are associated with these intracellular membranes. Arrows mark the characteristic tight junction membrane kisses. Scale bar, 100 nm.

Examination of T84 tight junction domain shows many interesting and novel features that have not been reported in the literature before. One of the striking observations was the apparent attachment of cytoskeletal bundles end-on to the submembrane domain of the tight junction (Fig. 11A). The actin cytoskeleton has been seen to localize to the tight junction in detergent extracted and myosin S1 decorated samples [3,70]. However, it is unclear whether these are end-on interactions or side-parallel interactions. The present images are the first examples of cytoskeletal attachment showing end-on interactions in non-extracted samples. These observations suggest that the tight junction of T84 cells contains proteins that allow end attachment of cytoskeletal bundles, perhaps even machineries to stimulate actin polymerization at specific membrane sites. Another striking observation from these images was that the tight junction membrane appeared to be pulled into one cell by the cytoskeletal bundles from the adjacent cell, suggesting that mechanical force was applied. Thus, a force generating apparatus consisting of a motor may be involved at these end-on cytoskeletal-tight junction interaction areas.

The second distinctive feature of T84 cells was the presence of intracellular membranes in various sizes and shapes around the tight junction cytoplasmic domain (Fig. 11B). Dense particles were prevalent in association with these structures. Interestingly, the membrane kisses of tight junctions frequently extended down the apical-lateral membrane for over half a micron whenever there were pools of associating intracellular membranes. These observations suggest that some regions of T84 tight junctions may be under continuous remodelling. Alternatively, the intracellular membranes may be permanently attached to the tight junction domains to regulate local activities such as calcium release from intracellular stores. Therefore, the tight junction is likely to contain linker proteins that allow tethering of intracellular membranes as well as machineries that regulate membrane dynamics.

**Figure 12:**
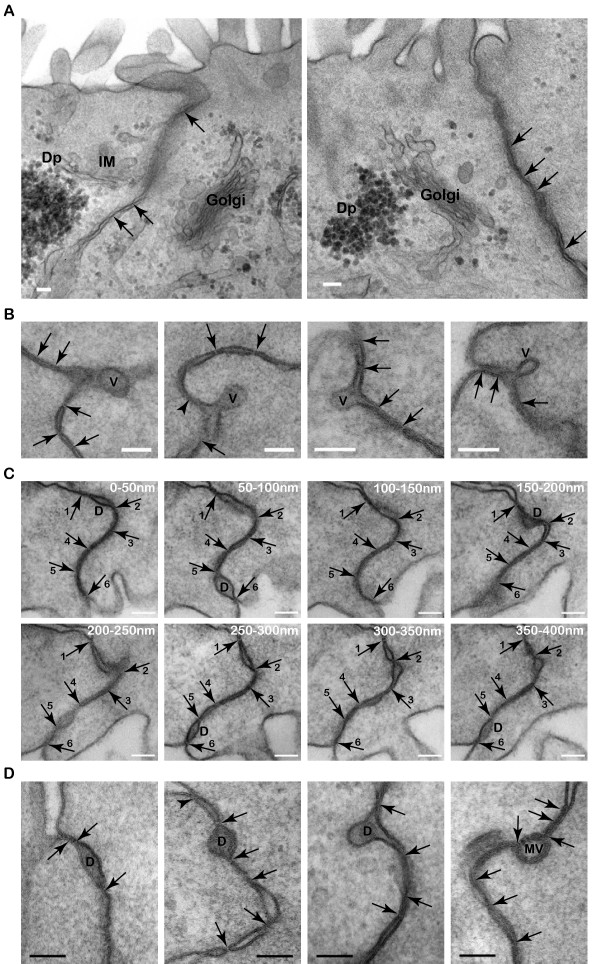
**Membrane processes associated with the tight junction**. (A) Satellite Golgi apparati are located 0.5 μm away from tight junction membranes. Arrows mark the characteristic tight junction membrane kisses. Intracellular membranes, IM, and numerous dense particles, Dp, are closely associating with the Golgi. Scale bar, 100 nm. (B) Small vesicles, v, with electron dense materials docked or fusing/budding within the tight junction membrane kisses. Arrows mark the characteristic tight junction membrane kisses. Scale bar, 100 nm. (C) Serial 50 nm sections showing discreet small pockets formed between tight junction kisses with electron dense material, D. Numbered arrows mark the respective tight junction kisses in consecutive sections. Scale bar, 100 nm. (D) Electron dense material, D, and membrane vesicles, MV, are found between the characteristic tight junction membrane kisses, arrows. Scale bar, 100 nm.

The third striking feature of T84 cells was the presence of satellite Golgi apparatus at the proximity of the tight junction (Fig. 12A). This observation suggests that the tight junction may be regulated locally and dynamically through satellite protein synthesis machineries. In addition, small vesicles with electron dense materials were seen in transit within the tight junction membrane domain (Fig. 12B), suggesting that the tight junction might be an area of intercellular communication via targeted local secretion. Thus, receptors and signaling molecules are expected to localize at these tight junction corralled domains where secretions occur. Proteins that are involved in vesicular trafficking including docking and fusion/budding of small vesicles are also expected to localize within the tight junction membrane domain.

The fourth distinctive feature of T84 tight junction was the presence of electron dense transient pockets within the kisses of the tight junction (Fig. 12C & D). Serial sections at 50 nm thickness showed that the pockets were frequently less than 100 nm in diameter (Fig. 12C). These extracellular materials may be the result of targeted vesicular secretion, which can act as messengers in a juxtacrine manner to regulate activities of neighbouring cells.

**Figure 13:**
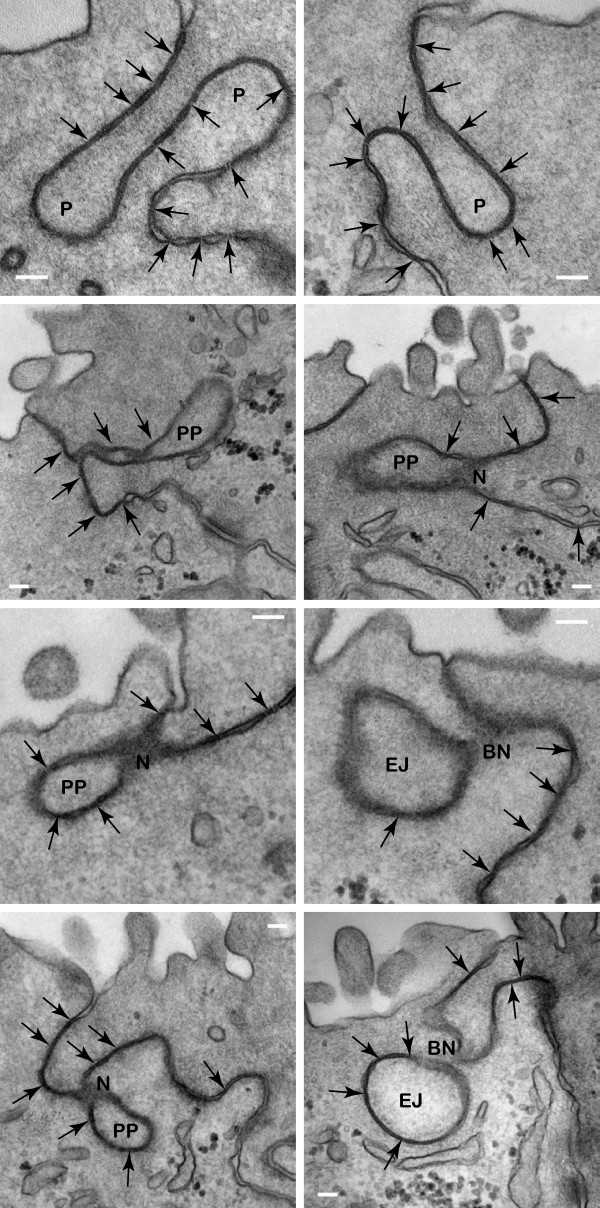
**Endocytosis of the tight junction**. Protruding fingers, P, are formed between neighbouring cells at the tight junction. Pinching protrusions, PP, of double plasma membranes from opposing cells are frequently associated with necks, N, that contain electron dense materials. Endocytosed junctions, EJ, have double plasma membranes and broken necks, BN. Arrows mark the characteristic tight junction membrane kisses. Scale bar, 100 nm.

One of the most interesting features of T84 tight junction was endocytosis of the double membranes (Fig. 13). The endocytosis of tight junction appeared to be preceded by invaginations/protrusions of the double membranes. Subsequently, the invaginations were pinched at the neck regions with associating electron dense materials. Finally, the double membranes were seen completely broken off from the surface plasma membrane and endocytosed into one cell. Several previous studies have tried to address the question of tight junction turnover [71-73]. Endocytosis of GFP-claudin has been observed during intercellular movement of EPH4 mammary epithelial cells [71]. Under non-physiological treatment with latrunculin A, occludin has been found to be endocytosed via a caveolin-mediated process [72]. On the other hand, a clathrin-mediated process is used for endocytosis of tight junction proteins during non-physiological removal of extracellular calcium [73]. However, the present images are the first examples of tight junction endocytosis in unperturbed confluent and polarized cells. These observations indicate that the double membranes are capable of creating membrane curvatures to form invaginations/protrusions between 2 adjacent cells. This specialized process is likely to require a novel set of proteins that can facilitate the fusion and fission of double membranes, which is possibly unrelated to the conventional clathrin-mediated or caveolin-mediated processes.

### Conceptualization of proteomics and bioinformatics results

**Figure 14:**
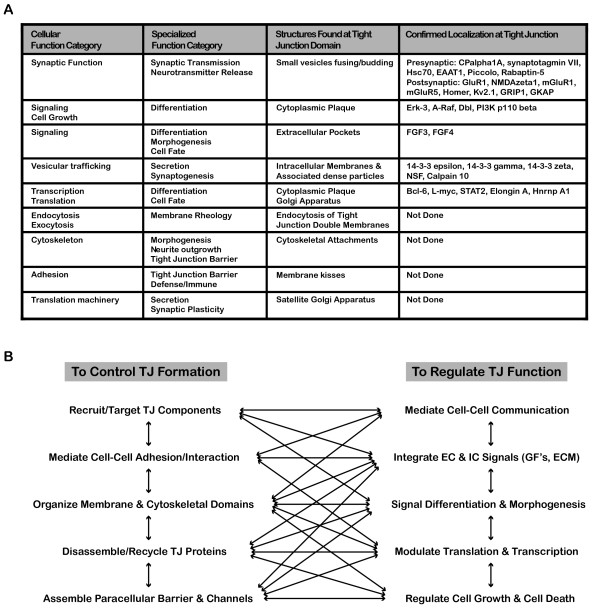
**Conceptualization of hits in the context of tight junction functions**. (A) Binning cellular functions and specialized processes with tight junction structures. (B) Complexity and interdependency of different processes involved in the formation and function of the tight junction.

Most of the strong clusters in both the cellular function and specialized process categories could be correlated with ultrastructures that were observed within the tight junction domain (Fig. 14A). Synaptic function could be correlated with the observations of small vesicle fusing/budding within the tight junction kisses. Messengers and signaling function could be correlated with extracellular pockets and cytoplasmic plaque. Vesicular trafficking function could be correlated with the arrays of intracellular membranes of different shapes and sizes. Translation function could be correlated with satellite Golgi apparatus and the associated dense particles.

Cytoskeletal function could be correlated with the observed cytoskeletal bundles attached to tight junction membranes. Adhesion function could be related to the membrane kisses and the close proximity of the apposed plasma membranes. In summary, the tight junction could be divided into multiple sub-domains to carry out localized functions and processes.

The complexity and inter-dependency of tight junction functions and processes can be outlined in a working model (Fig. 14B). Tight junction proteins could either participate in the formation of the tight junction or help to regulate the various cellular functions associated with it. These processes are functionally linked to each other such that perturbation in one process will affect the other processes.

## Discussion

### PART 1 – pros and cons of biochemistry, proteomics, & bioinformatics

Biochemical purification of membrane components remains a challenge to cell biologist because the procedure necessitates the use of detergents, which undoubtedly disrupt weak and transient protein complexes. This study provides a starting point for future improvements on fractionation and isolation procedures of membrane protein complexes from pure source of cultured cells, especially epithelial cells. The method also provides a starting point for optimization of large-scale production and purification of membrane proteins expressed in heterologous systems.

A total of 912 hits have been found in this study, a number that is likely to increase with time as better detection methods are developed. Furthermore, different epithelial cells are expected to have distinct tight junction proteomes because different epithelial cells can have different junctional structures [6,74] as well as expression of different tight junction proteins [75,76]. Out of the 912 hits, 20 are known tight junction components. There are several reasons for a low number of known tight junction proteins. First, many of the known proteins do not express in the same cell at the same time. Second, the estimate of 50 tight junction proteins is largely overwhelmed by 21 claudins, which are differentially expressed. Third, proteins with multiple transmembrane regions and post-translational modifications are very hard to detect due to the lack of identifiable peptides. For example, occludin and claudin-1 are clearly enriched when assessed by western blots but do not make the cutoff during scoring even though a few peptides have been found. The reasons are likely related to the facts that occludin has 4 transmembrane regions with at least 5 phosphorylation sites and claudins have 4 transmembrane regions with glycosylation and phosphorylation sites.

The tight junction preparation appeared to be relatively pure because none of the 912 hits belonged to other cell-cell junctions, such as E-cadherin of the adherens junction or desmosomal cadherin of the desmosome. This could be attributed to the unique and most important step of the protocol, which was prolonged swelling of cells while they were still attached on dish. The extreme distention of the plasma membranes permitted efficient fragmentation during subsequent homogenization. Another important step of the protocol was the employment of zwitterionic detergent CHAPS, which successfully solubilized tight junction complexes from plasma membranes and at the same time preserved interactions of tight junction components. However, it was difficult to assess whether all of the 912 hits are *bona fide *tight junction proteins. Based on previous studies, many known tight junction proteins have dual localization. For example, ZO-1 [77], ZO-2 [78], huASH1 [79] and symplekin [45] are localized to both the tight junction and the nucleus. Therefore, studies performed in non-polarized or non-epithelial cells are not helpful in determining whether the hits are *bona fide *tight junction proteins. Eventually, confirmation will have to be done one at a time using conventional methods such as immunolocalization and exogenous expression of tagged proteins in polarized epithelial cells.

### PART 2 -compare and contrast tight junction and synapse

The presence of synaptic proteins was a very surprising finding. However, there are noteworthy functional and structural features shared between the tight junction and the synapse. First, both structures contain satellite Golgi apparatus for local protein synthesis, which is important for plasticity of the synapse [80,81]. Second, both the tight junction and the synapse contain intracellular membranes of irregular shapes and sizes which are characteristic features of nerve terminals during synaptogenesis [82]. Third, both structures are functional and structurally linked to the cadherin and nectin-based adhesion systems [83-88]. Fourth, both the tight junction and the synapse are regulated by the Eph-ephrin cell-cell signaling system [89-93]. Fifth, both structures are intimately linked to the actin cytoskeleton in their formation as well as their normal function [94,95].

There are also many molecular similarities between the tight junction and the synapse. First, both structures have prominent representations of membrane associated guanylate kinase MAGUK proteins at their submembrane scaffolds [39,96-98]. In addition, MUPP1, a multi-PDZ MAGUK protein of the tight junction, can form synaptic complexes and regulates synaptic plasticity at the glutamatergic terminal [99-101]. Second, MAGI-2, another multi-PDZ containing protein of the tight junction [102], can also form synaptic complexes with AMPA receptors and its regulator stargazin in the brain [103]. Interestingly, stargazin belongs to the claudin superfamily of tight junction associated proteins and has been shown to mediate cell-cell adhesion similar to claudins [104]. Third, members of the exocyst complex sec6/8 that regulate vesicle delivery are localized to both the tight junction and synaptic membranes [105-109]. Fourth, the small GTP-binding protein rab3B that regulates calcium-dependent exocytosis and synaptic vesicle release has also been shown to present at the tight junction [110-112]. Fifth, the VAMP-associated protein VAP-33 that functions in neurotransmitter release also localized to the tight junction and binds occludin in epithelial cells [113,114].

Comparison of the postsynaptic proteome databases with the current list of hits showed striking similarities in the functional distribution of proteins, including 20% in signaling, 10% channels and transporters, and 15% in adhesion and cytoskeleton, and 10% in translation and transcription [115,116]. In addition, the total number of proteins identified in the synapse proteome is about 1100, which is comparable to the 912 hits found in this study.

The finding that tight junctions of epithelial cells contained a major cluster of synaptic proteins further supports the role of extra-neuronal synaptic proteins in non-neuronal cells [117-119]. For example, synaptic vesicles have been found in many non-neuronal cells including pinealocytes [120], chromaffin [121,122] and pancreatic beta cells [123]. Receptors for the excitatory neurotransmitter glutamate have also been found in non-neuronal cells including retinal pigment epithelial [124], urinary bladder epithelial, prostate epithelial cells [125] as well as osteoblasts [126-129]. Furthermore, glutamatergic NMDA receptors have been shown to localize to cell-cell contacts and participate in regulation of cell growth, differentiation, and migration in keratinocytes [130,131]. Interestingly, studies of polarized trafficking in osteoblasts indicate localization of tight junction proteins occludin and claudins at their intercellular contacts. These intercellular contacts appear to be the major target sites of vesicular traffic that involves synaptic components [132]. Indeed, osteoblasts have been shown to have targeted vesicular release of glutamate [133] that acts on NMDA receptors to regulate bone formation and resorption [129,134]. Generation of action potential has also been shown in several non-neuronal cells including pituitary endocrine [135], adrenocortical [136], pancreatic beta [137], osteoblastic [138], as well as chromaffin cells [139]. Furthermore, regenerative action potentials have been observed in chromaffin cells [140] and pigmented ciliary body epithelial cells [141], suggesting that a synaptic mechanism of propagation similar to neurons may present in these non-neuronal cells.

### PART 3 – new frontiers for tight junction biology

Beside synaptic transmission, differentiation and morphogenesis were also found as strong clusters in the specialized process category, suggesting that the tight junction might regulate cellular activities of the entire epithelial monolayer. Indeed, there were prominent clusters of transcription and translation in the cellular function category that might have roles in differentiation and morphogenesis. Clusters in secretion could partake in signaling events during differentiation; clusters in cytoskeleton could partake in force generation during morphogenetic cell movements.

Migration of intestinal epithelial cells is regulated by calcium-activated RhoA activity, which depends on the influx of extracellular calcium [142]. The influx of calcium is, in turn, controlled by membrane potential dictated by ion channels on the plasma membrane [143]. Indeed, membrane depolarization has been shown to regulate cell migration and epithelial wound healing [144]. This is interesting because both voltage-gated calcium and voltage-gated potassium channels were among the 912 hits in this study, suggesting that the tight junction may be able to regulate cell migration via these channels. Furthermore, calcium can also come from intracellular stores through inositol trisphosphate receptors. Indeed, inositol trisphosphate receptors have been localized to tight junctions of MDCK [145] and salivary acinar cells [146]. Therefore, it appears that the tight junction have multiple mechanisms to increase local concentration of calcium which may have roles in calcium-activated RhoA activity and cell migration. Interestingly, during dynamic epithelial cell movements, the local level of RhoA has been found to be regulated by PKC zeta at cell protrusion sites [147]. In addition, a PKC zeta-interacting protein, ZIP3, has been shown to bind to ion channels, further supporting a potential molecular link between PKC zeta and plasma membrane channels during cell movements [148].

## Conclusion

The striking similarities in molecular, structural, and functional features between the tight junction and the synapse suggest that epithelial cells may utilize the same proteins that neurons use for synaptic transmission to perform a similar function. However, there are also many molecular, structural, and functional differences between epithelial tight junctions and neuronal synapses that distinguish these very different cell types. For example, it is not known whether epithelial cells in the intestine can propagate action potential from one cell to another. It is also not known whether epithelial synaptic proteins function to regulate cell-cell communication or other activities at the plasma membrane. However, the structure within the tight junction domain appears to be an active zone of vesicular trafficking. In addition, the presence of claudins and PMP22 at both the tight junction and myelin suggests that these proteins may form an electrical insulation at the tight junction similar to their function in myelin. An alternative name, zonapse, is proposed here to incorporate the synaptic and zonula characteristics of the epithelial junction. The new nomenclature is meant to help distinguish the diverse roles of tight junction proteins because some of the proteins will have the classical "tight junction" functions such as formation of permeability barrier and maintenance of membrane polarity whereas other proteins will have "zonaptic" functions such as cell-cell communication via release of transmitters and propagation of intercellular signals.

The presence of zonapse at the apical domain would allow cells to communicate through secretion of transmitters within the confined extracellular space delineated by the tight junction membrane barrier, which could directly stimulate or inhibit activity of neighbouring cells. Such signals when propagated and amplified via the network of tight junctions within a monolayer of epithelium may spread one-dimensionally or two-dimensionally depending on the expression patterns of the pre-zonaptic and post-zonaptic components within the epithelium. This would definitely provide a novel way of looking at epithelial sheets as two-dimensional organs, which is essential for understanding complex processes such as epithelial differentiation and morphogenesis where a group of cells must behave in a coordinated manner. For example, intestinal epithelia are turned over continuously and replenished by proliferation of stem cells located close to the base of crypts. Many different cell types are differentiated from stem cells; some move up the crypt-villus axis to become surface absorptive epithelial cells and some move down to become secretory cells at the base of the crypt. However, it is unclear how the progenitor cells coordinate among themselves to differentiate and migrate along the crypt-villus axis to their final destinations. One possibility is that the cells are constantly communicating with each other and the entire epithelium behaves as a unit. Thus, analysis of zonaptic function may provide insight into regulation of fundamental processes such as regeneration and maintenance of the intestinal epithelium.

## Methods

### Cell culture

T84 (from ATTC) cells were maintained in DMEM/F-12 supplemented with 10% fetal bovine serum. MDCKI (from Kai Simons, EMBL) cells were maintained in MEM with Earle's salts supplemented with 10% fetal bovine serum. C2bbE1 (from ATTC) cells were maintained in DMEM supplemented with 10% fetal bovine serum, insulin, transferrin, and selenium. Cells were grown in cell culture dishes or in Transwell-clear inserts (Costar, Corning).

### Biochemistry for proteomic analysis

T84 cells (15 × 150 mm dishes) were plated at 100% confluent density at 18 hours before utilization for biochemistry. On the first day of purification, cells were quickly rinsed four times with ice cold 10 mM HEPES, pH 9.0, and incubated in the same buffer for three hours at 4°C without agitation. At the end of three hours, in the cold room, the buffer was decanted quickly and gently to avoid sloughing off of cells from the plate.

To maintain the highest protein concentration possible in the initial cell lysate, the remaining buffer was removed as much as possible by tilting the plate and using a pipetman. Cell were immediately scraped off the plates and collected in a 50 ml conical tube chilled on ice. After all the cells were collected from the plates, they were passed 10 times through a 22 G needle. Efficiency of homogenization was examined under phase-contrast of trypan blue stained whole cell lysate. After homogenization, protease inhibitors (Antipain, 50 μg/ml; Aprotinin, 2 μg/ml; calpain inhibitor I, 17 μg/ml; calpain inhibitor II, 7 μg/ml; E-64, 10 μg/ml; leupeptin, 1 μg/ml; Pefabloc SC, 1 mg/ml; pepstatin A, 1 μg/ml; Roche Molecular Biochemicals) were added immediately with continuous vortex. Large organelles, nuclei, and whole cells were removed by centrifugation at 30,000 g for 10 minutes at 4°C. The supernatant was centrifuged at 100,000 g for 60 minutes at 4°C onto a saturated sucrose cushion. Membranes were collected at the buffer and sucrose interface, washed with ice-cold distilled water, and re-centrifuged at 100,000 g onto a saturated sucrose cushion in a new tube. The washed membranes were collected at the water/sucrose interface and diluted in 10% CHAPS in distilled water (chilled to 4°C) to a final concentration of 2% CHAPS. The membranes were then incubated with rabbit anti-PKC zeta antiserum at 5 μL per 150 mm dish of cells (P0713, Sigma) or control anti-serum for 18 hours at 4°C. Membranes were centrifuged at 10,000 g for 30 min at 4°C to remove precipitates. Cleared supernatants were incubated with Protein A-Sepharose beads at 80 μL of beads per 150 mm dish of cells (Sigma) for 18 hours at 4°C. Beads were washed 6 times with ice cold 2% CHAPS in distilled water. PKC zeta immuno-complexes were eluted with a PKC zeta peptide antigen (KGLYINPLLLSAEESV, Research Genetics, Inc) in 100 mM NaCl, 15 mM HEPES, pH 7.5, for 60 min at 25°C. A small fraction of the eluted immuno-complex was used immediately for negative stain electron microscopy. The rest of the sample eluate was passed through a 25G Sepharose spin column to remove salts and subsequently concentrated about 5 fold using a speedvac. The concentrated samples were either used immediately for gel analysis or stored at -80°C.

### Biochemistry for heavy plasma membranes

T84 cells (15 × 150 mm dishes) were grown to 100% confluent density and maintained for one week post-confluency before utilization for biochemistry. Cell homogenate was obtained using the same protocol as for proteomics (see above). In addition to protease inhibitors (see above), dithiothreitol (DTT) was added to a final concentration of 10 mM to the cell homogenate. Large plasma membrane sheets were obtained by centrifugation of cell homogenate at 1,000 g for 15 minutes at 4°C. The pellet was resuspended in 10 mM Hepes/10 mM DTT/pH8 with a 20G needle. The resuspended membranes were loaded onto a sucrose step gradient of 15%w/v–37%w/v–47%w/v–60%w/v and centrifuged at 100,000 g for 5 hours at 4°C. Membranes at the interfaces of sucrose steps were concentrated by re-centrifugation at 100,000 g onto a saturated sucrose cushion in a new tube. The fraction at 47%w/v–60%w/v interface was found to enrich in tight junction protein occludin.

### SDS-PAGE, western blots, and silver stain

For SDS-PAGE, protein samples were solubilized to a final concentration of 2% SDS/2 mM EDTA/20 mM Tris pH 6.8. The samples were boiled for 15 minutes and cooled to room temperature before loading onto the gel. For silver staining of polypeptides, the eluted proteins were resolved with 4–15% SDS-PAGE, fixed in 50% methanol for 30 min, rinsed in 5% methanol, reduced in 10 mM DTT in distilled water for 30 min, stained with 5% silver nitrate in distilled water for 15 min, and developed immediately in 100 mM sodium carbonate/0.375% formaldehyde. The stained gel was transferred quickly to a new vessel containing 10% acetic acid to quench the silver staining reaction. For western blot analysis, proteins were transferred to nitrocellulose paper (0.45 μm, Biorad). The blots were pre-incubated in 150 mM NaCl/20 mM HEPES pH7/0.1% TX-100/5% non-fat dry milk for 2 hours before probing with anti-occludin (33–1500, Zymed), anti-claudin-1 (71–7800, Zymed), anti-ERK3 (sc-156, Santa Cruz Biotech), anti-STAT2 (sc-476, Santa Cruz Biotech), anti-Homer (sc-15321, Santa Cruz Biotech), anti-Hsc70 (sc-7298, Santa Cruz Biotech), anti-mGluR5 (Ab27190, Abcam), anti-connexin 36 (sc-14904, Santa Cruz Biotech), anti-EAAT1 (sc-15316, Santa Cruz Biotech). Blots were washed 3 times and probed with HRP-conjugated secondary antibodies (anti-mouse HRP #170–6516, Biorad; anti-rabbit HRP 170–6515, Biorad; anti-goat HRP #81–1620, Zymed). After washing 5 times, the blots were developed using a ECL kit (#94–0144, Amersham) and images were captured on film. All images were digitally scanned and imported into Adobe Photoshop for figure preparation. No digital manipulation has been done to any images.

### Mass spectroscopy, fingerprint analysis, and bioinformatics

For mass spectroscopy analysis, eluted proteins were solubilized to a final concentration of 2% SDS/2 mM EDTA/20 mM Tris pH 6.8. The samples were boiled for 15 minutes and cooled to room temperature before loading onto the gel. Proteins were separated with 4–15% SDS-PAGE, stained with 0.2% commassie blue/20% methanol/10% acetic acid, de-stained with 20% methanol/10% acetic acid, and stored in 10%acetic acid. All visible bands were excised (supplement figure S2) and submitted for in-gel tryptic digestion and MALDI-TOF mass spectroscopy analysis (Molecular Biology Core Facilities, Dana-Farber Cancer Institute). Peptide masses of the same identifiable bands from three separate experiments were combined for fingerprint analysis (supplement figure S2). All searches were done using MS-fit peptide mass fingerprint tool from the UCSF Mass Spectroscopy facility (Protein Prospector, UCSF) on SwissProt database (2005.01.06) with mass tolerance of 50 ppm, maximum number of missed cleavage of 1, and protein/peptide modifications (peptide N-terminal Gln to pyroGlu, oxidation of M, and protein N-terminus acetylation). All candidates were individually scored and the final list of hits entered into a tight junction proteomics database using a customized filemaker table (see Supplement figure S2). Parameters used for scoring and bioinfomatic analysis were obtained from Swiss UniProt and PubMed. Bioinformatics analysis was performed using a customized filemaker table (supplement figure S3).

### Immunofluorescence

Cells were grown to confluence on Transwell-clear inserts (Costar). C2bbE1 cells were 4 weeks post-confluent with well-formed tight junctions and transepithelial electrical resistance of 400-ohm cm square. MDCK I cells were 10 days post-confluent with well-formed tight junctions and transepithelial electrical resistance of ~10,000-ohm cm square. Before fixation, cells were chilled at 4 degree Celsius for 3 hours. Cells/Transwells were rinsed five times in 150 mM NaCl and 10 mM HEPES, pH 7.5 at 4°C, and immediately fixed in 100% methanol at -20°C for a minimum of 18 hours. Subsequently, the cells/Transwells were rinsed briefly (30 seconds) in 100% Acetone at -20°C and allowed to air-dry at room temperature. Cells/Transwells were used immediately or stored at 4°C until use. For immunofluorescence staining, cells/filters were excised with a razor bald and the filters were transferred to a multiwell plate. Cells/filters were rinsed twice with 150 mM NaCl and 10 mM HEPES, pH 7.5. Primary antibodies were incubated overnight at room temperature in the same buffer. Antibodies to P/Q-type calcium CP alpha 1A (sc-28619), synaptotagmin VII (sc-15420), EAAT1 (sc-15316), piccolo (sc-18569), connexin 36 (sc-14904), GRIP1 (sc-28934), rabaptin-5 (sc-6162), Homer (sc-15321), NMDA zeta (sc-1467), GluR1 (sc-13152), Hsc70 (sc-7298) were purchased from Santa Cruz Biotechnology. Antibodies to metabotropic glutamate receptor 1 (G7794) were purchased from Sigma. Antibodies to metabotropic glutamate receptor 5 (ab27190) were purchased from Abcam. Antibodies to Kv2.1 (AB5186) were purchased from Chemicon. An antibody to occludin (33–1500) was purchased from Zymed. After primary antibodies incubation, cells/filters were washed 3 times with 150 mM NaCl and 10 mM HEPES, pH 7.5 and incubated with secondary antibodies (FITC and Cy3 conjugated secondary antibodies; anti-mouse AP160F, AP124F & AP192F, anti-goat AP106C & AP180C, anti-rabbit AP156C & AP132F from Chemicon) for 3 hours at room temperature, washed 6 times, and post-fixed with 100% ethanol at -20°C. Cells/filters were air-dried at room temperature and mounted immediately onto slide using ProLong Gold anti-fade (Molecular Probe) and coverslips. Confocal images were obtained from Biorad2000 attached to Nikon E800 using Lasersharp imagine acquisition software (Nikon Imaging Center, Harvard Medical School). All images were imported into Adobe Photoshop for preparation of figures. No digital manipulations have been done to any images.

### Negative stain transmission electron microscopy

Samples were layered onto formvar coated/carbon coated/glow discharged copper grids and allowed to sit for 2–5 min. The grids were rinsed 3 times with water and stained with 2% uranyl acetate for 1–3 min. After removal of excess uranyl acetate solution, the grids were air-dried and examined at 60 kV in JEOL-1200EX. Images acquired on negatives were scanned and imported into Adobe Photoshop for figure preparation.

### Ultra-thin section transmission electron microscopy

T84 cells were plated at 15% confluent density and allowed to grow to confluency for 10 days. Cells/Transwells were chilled at 4°C for 6 hours before fixation with 3.75% glutaraldehyde, 150 mM NaCl, 20 mM HEPES, pH 7.5 at 4°C for 18 hours. The fixation reaction was quenched with 50 mM glycine, 150 mM HEPES, pH 7.5 on ice for 1 hour. Cells/Transwells were rinsed in ice cold distilled water for 3 times, secondary fixed with 1% osmium tetroxide/1.5% potassium ferrocyanide for 2 hours on ice, rinsed 4 times in ice cold distilled water, en bloc stained with freshly prepared and filtered 2% uranyl acetate in distilled water on ice for 2 hours, and rinsed 4 times in ice cold distilled water. Cells/Transwells were dehydrated with sequential 5-minute incubations in 50%, 75%, 95%, 100%, 100%, 100% ethanol at room temperature. Epon-Araldite (EMbed 812) was added to Transwells and allowed to polymerize at 60°C for 48 hours. Ultrathin sections were cut using a Reichert Ultracut-S microtome, layered onto carbon coated copper grids, and stained with freshly made/filtered 2% lead citrate. Grids were examined with Tecnai G2 Spirit BioTwin equipped with an AMT 2 k CCD camera. Digital images acquired were imported into Adobe Photoshop for figure preparation.

## Competing interests

The author(s) declare that they have no competing interests.

## Authors' contributions

V.T. planned and performed all experiments, data analysis, and preparation of the manuscript.

## Reviewer's comments

### Reviewer's report 1

Gáspár Jékely, European Molecular Biology Laboratory

This paper presents a novel purification protocol and a proteomic approach to identify several novel tight-junction components. Many of the identified proteins are synaptic proteins hinting at an unexpected functional similarity of the two structures. The paper also presents a detailed EM study of tight junctions and describes interesting morphological features.

It is an impressive and important work but a more extensive discussion and some reorganisation of figures and text would greatly increase its value. In contrast to the amount of new and interesting data the paper seems to be quite concise, a more lengthy discussion of the results is needed. We don't read anything about how the proteomic analysis could help us to understand the unique features of tight junctions listed in the introduction and the morphologies described by EM. At least a tentative link should be made between some of the novel components and some of the described features/morphologies. For example several endocytic proteins (e.g. rabaptin-5, Hsc70) were identified and localised in punctate structures at the tight junction that could be responsible for regulating tight junction endocytosis. What may be the role of synaptic signalling proteins in cell-cell communication? What about adhesion, signalling and the links to the cytoskeleton? What are the novel tight junction components and what is there role in other cellular processes?

Authors's response: The manuscript has been substantially re-written. The section showing EM pictures has been re-written (Fig. 11, 12, 13) to discuss novel processes within the tight junction found in this study. A table has been added (Fig. 14A) to correlate EM structure and function. A comprehensive Introduction, and expanded Discussion & Conclusion sections have been included.

The quality of the preparations subjected to mass-spec analysis is carefully checked by EM, yet one is left wondering about the purity of the preps and the specificity of the hits. Although several hits were verified by immunostainings, there is no general overview: we don't know how many antibodies were tried and how many gave positive or negative results. Was it a randomised test or only TM proteins were checked? One could imagine for example that some cytoskeletal components that are otherwise not enriched at the tight junction co-purify through links to actin cables that bind to tight junction components (e.g. cadherin).

Authors's response: A paragraph has been added to the Introduction to discuss previous biochemical methods and identification of tight junction proteins. A paragraph has been added to the Discussion to address the question of purity and specificity. A supplementary figure S1 has been added to show co-enrichment of a few of the new proteins with the tight junction marker occludin. A table has been added (Fig. 14A) to show confirmation of hits other than synaptic proteins. A list of antibodies in this study has been added as a supplementary figure.

### Reviewer's report #2

Etienne Joly, Equipe de Neuro-Immuno-Génétique Moléculaire, IPBS, UMR CNRS 5089

This very interesting paper is likely to represent a very significant advance in understanding the biology of tight junctions (TJ). The initial step is the development of a novel protocol to purify tight junctions from a human epithelial cell in culture. This is then used to undertake a proteomic characterisation of the TJ components, yielding an impressive list of 912 hits. Quite remarkably, bio-informatic analyses of the proteomic results reveals that there is a very significant overlap between the protein constituting TJ and those previously identified as constituent of neuronal synapses. This raises the intriguing possibility that TJ and synapses may share certain similar functions.

### Reviewer's report 3

Neil Smalheiser, University of Illinois at Chicago

#### Overview

This is an interesting and significant paper that presents, for the first time, a method of purifying tight junction complexes. The tight junctions are unexpectedly enriched in synaptic and signaling proteins, suggesting that they may be involved in cell-cell signaling processes. The paper needs revising because it gives too much room to irrelevant or redundant findings; at the same time, it gives too little room to discussing critical technical details, as well as the broader context and physiologic implications.

Authors's response: The manuscript has been substantially re-written. The number of figures has been reduced to 14 (plus 3 supplementary figures). A comprehensive Introduction, and expanded Discussion & Conclusion sections have been included.

#### Results and discussion

I don't see the relevance of showing EM pictures of cultured T84 cells (fig. 2, 3, 4, 5, 6, 7, 8) – how is this novel or how does it support the data in the paper?

Authors's response: The section showing EM pictures has been re-written (Fig. 11, 12, 13) to discuss novel processes within the tight junction found in this study. A table has been added (Fig. 14A) to correlate structure with function.

Are there any previously described methods for purifying tight junction complexes at all? If so, they should be cited and discussed.

Authors's response: Yes, one study in over 20 years. A paragraph has been added to the Introduction to discuss previous biochemical methods and identification of tight junction proteins.

The immuno-purification protocol is not precisely described. What was the control antibody? Pre-immune serum? 

Authors's response: Control serum was discussed in the Methods section in the previous manuscript. 

The best control would be an antibody that brings down a different cellular compartment, i.e. that is positively enriched in some proteins but not those found in the tight junctions. 

Authors's response: Unless one is interested in comparative proteomics, comparison of the tight junction fraction with other cellular compartments does not speak to whether a particular protein is important in tight junction function. For example, Protein X could have function at the tight junction as well as other cellular compartments. A thorough discussion of the specificity and purity of the tight junction preparation has been included in the Discussion. 

Figure 11 shows discrepancies (missing lanes that are described in the legend but not found in the figure). 

Authors's response: Fixed.

The negative staining of isolated tight junction complexes could be shown in a single figure with a few panels, fewer than is currently shown (fig 11, 12, 13). Also, it is not very persuasive simply to show photos with no quantification.

Authors's response: Illustrations have been reconfigured and condensed.

The methods section is lacking in details such as antibody concentrations, and there is no verification that the antibodies used for immunofluorescence are actually specific for the desired proteins (e.g. by doing Western blotting). What, if anything, was used as a blocking solution during immunostaining? 

Authors's response: Details added to the Methods section. A list of antibodies and their concentrations used in this study has been added as a supplementary figure. 

It is important to show not only that occludin and PKC zeta are enriched in isolated tight junctions, but that some or all of the synaptic proteins are enriched biochemically in this fraction as well by Western blotting. 

Authors's response: A supplementary figure S1 has been added to show co-enrichment of a few of the new proteins with the tight junction marker occludin. 

Conversely, it is NOT important to show all of the primary photos taken for each cell line, for each antibody, in fig. 19-28, unless specific points are being made (e.g. punctuate staining patterns). 

Authors's response: Illustrations have been reconfigured and condensed.

Are any of the synaptic proteins localized to tight junctions in epithelial cells in vivo? Or is something that only occurs in cultured cells?

Authors's response: This is a very good and important question that I do not have an answer at this moment. I have tried to stain formaldehyde fixed human colon sections but none of the antibodies worked, not even the occludin antibodies. I think the tight junction area is very compact and inaccessible. For tissue culture cells, I had to fix the cells overnight in methanol and expose the antigens/epitopes by acetone denaturation before I can get the stainings to work. I believe one may have to express tagged proteins in transgenic animals or spend the time optimizing frozen sections for immunofluorescence. Alternatively, I am planning to try immunogold cryoEM, which provides higher resolution as well as better preservation of antigenicity.

The poor yield of known tight junction components in the MS profile may be due, in part, to the fact that the author first separated individual bands on a gel before subjecting them to proteolysis. If the entire junction were to be proteolyzed without any gel separation first, the yield might be much better. There no details given about how the proteins were separated on the gel – were they dissolved in SDS? (What concentration? Boiled? Under reducing conditions?)

Authors's response: Details added to the Methods section.

The author should discuss whether anything is already known about the function of these "synaptic proteins" in T84 or other epithelial cells. Is it surprising that they are expressed in epithelial cells per se, or are they well known to be expressed there? Have any epithelial cells been shown to exhibit regulated glutamate release? Have the glutamate receptors expressed on epithelial cells been shown to mediate biochemical responses that participate in any known physiologic process? I found at least two previous reports of various non-neuronal cell types that have functional synaptic vesicles and/or regulated glutamate release; these are not epithelial cell types but may be worth discussing in terms of the broader context that synaptic proteins have signaling roles in many cell types:

Authors's response: These are helpful suggestions. A new Discussion section has been added.

Rastaldi MP, Armelloni S, Berra S, Calvaresi N, Corbelli A, Giardino LA, Li M, Wang GQ, Fornasieri A, Villa A, Heikkila E, Soliymani R, Boucherot A, Cohen CD, Kretzler M, Nitsche A, Ripamonti M, Malgaroli A, Pesaresi M, Forloni GL, Schlondorff D, Holthofer H, D'Amico G. Glomerular podocytes contain neuron-like functional synaptic vesicles. FASEB J. 2006 May;20(7):976–8.

Bhangu PS, Genever PG, Spencer GJ, Grewal TS, Skerry TM. Evidence for targeted vesicular glutamate exocytosis in osteoblasts. Bone. 2001 Jul;29(1):16–23.

#### Conclusion

There should be a separate section of Discussion, and the Conclusions section should look ahead to discuss possible future experiments or investigations. The author should not refrain from making an explicit model of how synaptic proteins at tight junctions might work – e.g., glutamate is released from one cell in response to some dynamic event and acts upon a neighboring cell via glutamate receptors localized (and presumably amplified in their actions) to tight junctions. How could this be tested further? What might this contribute to intestinal epithelium function? What are the implications for physiology, health or disease?

Authors's response: These are helpful suggestions. A new Conclusion section has been added.

## Supplementary Material

Additional File 1 Identification and scoring of hits. (A) Bands from 3 separate purifications were used for mass spectroscopy (see Methods for details). (B) Peptides from the same bands were pooled for fingerprint analysis. Click here for file

Additional File 2 Filemaker prototype for bioinformatic analysis Click here for file

Additional File 3 Co-enrichment of hits with occludin. (A) Western blots of whole cell, WC, and heavy plasma membrane, HPM, showing co-enrichment of ERK3, STAT2, Homer, Connexin 36, and mGluR5 with occludin. EAAT1 and Hsc70 are also present in the heavy plasma fraction (see Methods for details). (B) Antibodies used in this study. Click here for file
