# Supplementary material for: Proteomic and bioinformatic analysis of epithelial tight junction reveals an unexpected cluster of synaptic molecules
Source: Biol Direct. 2006 Dec 8;1:37. doi: 10.1186/1745-6150-1-37 (PMC1712231; doi:10.1186/1745-6150-1-37)
Supplement: Additional File 1 — Identification and scoring of hits. (A) Bands from 3 separate purifications were used for mass spectroscopy (see Methods for details). (B) Peptides from the same bands were pooled for fingerprint analysis. [file 1745-6150-1-37-S1.pdf]

A

Major Bands (kDa)	Expt #T43 ★ (red)	Expt #T46 ★ (blue)	Expt #T47 ★ (green)
22		X	X
24		X	X
26		X	X
31		X	X
33		X	X
35		X	X
40		X	
42		X	X
50		X	
60		X	
66		X	X
70		X	X
85		X	X
100	X	X	X
120	X	X	X
160		X	
180		X	
240		X	
300		X	

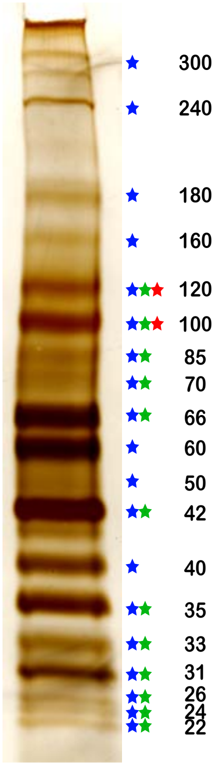

B

Search ID	Bands Used	Search MW (kDa)
22+	22, 24, 26	14-35
31+	31, 33, 35	22-47
40	40	30-55
42	42	33-55
50	50	40-65
60	60	45-80
66	66	50-80
70	70	50-90
85	85	65-125
100	100	75-145
120	120	85-160
160	160	130-260
180	180	150-240
240	240	180-300
300	300	240-700
