# Supplementary material for: Proteomic and bioinformatic analysis of epithelial tight junction reveals an unexpected cluster of synaptic molecules
Source: Biol Direct. 2006 Dec 8;1:37. doi: 10.1186/1745-6150-1-37 (PMC1712231; doi:10.1186/1745-6150-1-37)
Supplement: Additional File 2 — Filemaker prototype for bioinformatic analysis [file 1745-6150-1-37-S2.pdf]

Tight Junction Proteomics Hits

**Protein Name**	Voltage-gated P/Q-type calcium channel alpha-1A subunit
**Alternative Name**	Voltage-dependent calcium channel alpha subunit Cav2.1, Calcium channel L type, Alpha-1 polypeptide
**Accession Number**	O00555
**Family**	Calcium channel alpha-1 subunit
**Domains**	
**Binding Partner**	
**Configuration**	Integral Membrane
**Modulation**	☐ Lipid ☐ P ☐ Acetyl ☐ Ned ☐ Methyl ☐ ADP-R ☐ Sugar ☐ U ☐ Arg ☐ SUMO ☐ Sulfate
**Regulation**	☐ PI's ☐ mRNA Level ☐ IRES ☐ GTP ☐ ATP ☐ Splicing ☐ Ca ☐ RNA Editing ☐ PKC ☐ AMP ☐ cAMP ☐ Calpain
**Localization**	☐ Tight Junction ☐ Endosome ☐ IF Cytoskeleton ☐ Ectosome ☐ Centrosome ☐ Plasma Membrane ☒ Synapse ☐ Ribosome ☐ Hnrnp ☐ Nuclear ☐ Golgi/Golgi-drived ☐ Synaptic Vesicle ☐ Proteasome ☐ PARPs ☐ Cilia ☐ Actin Cytoskeleton ☐ Neurite ☐ Extracellular ☐ Cytosolic ☐ Mitochondria ☐ Intracellular Membrane ☐ ER/ER-derived ☐ Exosome ☐ Microtubule ☐ Unknown
**Molecular Function**	☐ Structural ☐ Adaptor ☐ GTPase ☐ Kinase ☐ Translation Machinery ☐ Receptor ☐ Catalyst ☐ GEF/GAP ☐ Phosphatase ☐ IRES-Machinery ☐ Messenger ☐ Modifier ☐ Peptidase ☐ Helicase ☐ Memb Fusion/Fission ☐ Transducer ☐ Chaperone ☐ Antiprotease ☐ Ribosomal ☐ Unknown ☐ Modulator ☒ Channel ☐ Transporter ☐ Splicing Factor ☐ Organizer ☐ ATPase ☐ Motor ☐ Transcription Factor
**Cellular Function**	☐ Signalling ☐ mRNA Stability ☐ Mitosis ☐ Translation ☐ Gene Silencing ☐ Cell-Cell ☐ Cytoskeleton ☐ Cytokinesis ☐ IRES-Translation ☐ RNA Silencing ☐ Adhesion ☐ Cell Migration ☐ Transcription ☐ Protein Processing ☐ RNA Editing ☐ Endocytosis ☐ Cell Protrusion ☐ RNA Processing ☐ Protein Degradation ☐ Unknown ☒ Exocytosis ☐ Cell Death ☐ Viral/Bacterial ☐ Energy/Metabolism ☐ Other... ☒ Synaptic ☐ Pro-Apoptotic ☐ DNA Replication ☐ Cell Homeostasis ☐ Vesicular Traffic ☐ Anti-Apoptotic ☐ DNA Repair ☐ Membrane Potential ☐ mRNA Targeting ☐ Cell Growth ☐ Biosynthesis ☐ Stress Response ☐ mRNA Turnover ☐ Cell Cycle ☐ Biodegradation ☐ Damage Control
**Signaling Pathway**	☐ 14-3-3's ☐ PLC ☐ PKCd ☐ Rab6 ☐ TGFb ☐ Formin ☐ KSR1 ☐ ACH ☐ 14-3-3b ☐ PLCb ☐ PKCep ☐ Rab27 ☐ TNFa ☐ Calpain ☐ Myc ☐ GABA ☐ 14-3-3e ☐ PLD ☐ PKCz ☐ Rho ☐ IL's ☐ COP9 ☐ Dab1 ☐ Glycine ☐ 14-3-3n ☐ PI3K ☐ Ras ☐ Rac ☐ Cytokine ☐ Shc ☐ PDK1 ☐ Glutamate ☐ 14-3-3g ☐ Erk ☐ Raf ☐ Cdc42 ☐ STAT1/2 ☐ Abl ☐ WT1 ☐ SCAR/WAVE ☐ 14-3-3t ☐ NO ☐ A-Raf ☐ PDGF ☐ Insulin ☐ Tec ☐ LKB1 ☐ Notch/Deltex ☐ 14-3-3z ☐ cAMP ☐ B-Raf ☐ VEGF ☐ AMPK ☐ ARF6 ☐ HSP90 ☐ Semaphorin ☒ Calcium ☐ AMP ☐ Rap1 ☐ FGF ☐ Serpin ☐ SHH ☐ Phox ☐ Caspase8 ☐ G Protein ☐ ATP ☐ Rap2 ☐ HGF ☐ p53/Myc ☐ Dbl ☐ Nck ☐ p53 ☐ Gaq ☐ PKA ☐ Rab ☐ EGF ☐ Wnt/Frz ☐ ApoB ☐ NFkB ☐ LIMK ☐ PI's ☐ PKB ☐ Rab3 ☐ IFNg ☐ GRP78 ☐ PP2A ☐ JNK ☐ PLA ☐ PKC ☐ Rab5 ☐ IFNa ☐ LFA-1 ☐ ATM ☐ 5HT
**Specialized Process**	☐ Metastasis ☐ Myelination ☐ RNA Polymerase II ☐ Synaptogenesis ☐ Glycolysis ☐ Inflammation ☐ Lf-Rt Asymmetry ☐ Synaptic Plasticity ☒ Secretion ☒ Communication ☐ Membrane Rheology ☐ Synaptic Translation ☐ Cell Fate ☐ Differentiation ☐ Cerebral Cortex Size ☒ Neurotransmitter Release ☐ G1/S ☐ Morphogenesis ☐ Tumor Suppression ☐ Neurotransmitter Synthesis ☐ G2/M ☐ Nutrient Uptake ☐ Actin Polymerization ☐ Glucose-Regulated Response ☐ Polarity ☐ Memory/Learning ☐ Leukocyte Migration ☐ Oxygen-Regulated Response ☐ TJ Barrier ☐ Wound Healing ☐ Paracrine Signalling ☐ Glutathione Homeostasis ☐ TJ Formation ☐ Respiratory Burst ☐ Insulin Release ☐ Asymmetric Cell Division ☐ Defense/Immune ☐ H2O2 Production ☐ Axon Guidance ☐ Organogenesis ☐ Stemness ☐ Iron Homeostasis ☐ Neurite Outgrowth
**Multifactor Disease**	☐ Cancer ☐ CMT ☒ Epilepsy ☐ Alzheimer's ☐ Schizophrenia ☐ Polycystic Kidney ☐ Diabetes ☒ Ataxia ☐ Deafness ☐ Huntington's ☐ Bowel Diseases
**Disease**	Familial hemiplegic migraine (FHM), Autosomal dominant spinocerebellar ataxia type 6 (SCA6), Episodic ataxia type-2 (EA-2, acetazolamide-responsive hereditary paroxysmal cerebellar ataxia APCA)
