# Supplementary material for: Proteomic and bioinformatic analysis of epithelial tight junction reveals an unexpected cluster of synaptic molecules
Source: Biol Direct. 2006 Dec 8;1:37. doi: 10.1186/1745-6150-1-37 (PMC1712231; doi:10.1186/1745-6150-1-37)
Supplement: Additional File 3 — Co-enrichment of hits with occludin. (A) Western blots of whole cell, WC, and heavy plasma membrane, HPM, showing co-enrichment of ERK3, STAT2, Homer, Connexin 36, and mGluR5 with occludin. EAAT1 and Hsc70 are also present in the heavy plasma fraction (see Methods for details). (B) Antibodies used in this study. [file 1745-6150-1-37-S3.pdf]

A

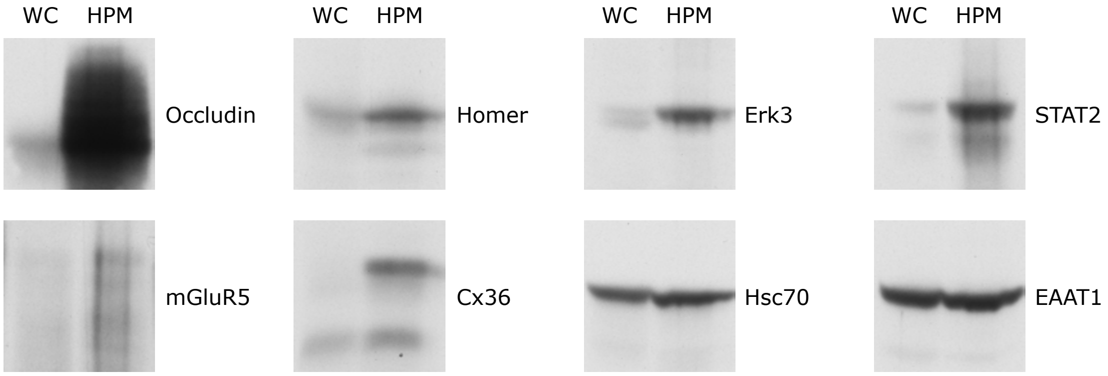

B

Protein	AB ID#	Antibodies	Specificity	Antigen	Concentration for staining	Concentration for blotting
Connexin 36	Sc-14904	Goat polyclonal	Affinity purified	Human C-terminus peptide	10 ug/ml	1 ug/ml
CP alpha 1A	Sc-28619	Rabbit polyclonal	IgG fraction	Human C-terminus 2225-2314	10 ug/ml	Not Done
EAAT1	Sc-15316	Rabbit polyclonal	IgG fraction	Human N-terminus 1-50	10 ug/ml	1 ug/ml
Erk3	Sc-156	Rabbit polyclonal	Affinity purified	Rat internal peptide	10 ug/ml	1 ug/ml
GluR-1	Sc-13152	Mouse monoclonal	IgG(1)	Human N-terminus peptide	10 ug/ml	1 ug/ml
GRIP1	Sc-28934	Rabbit polyclonal	IgG fraction	Rat C-terminus 910-1100	10 ug/ml	1 ug/ml
Homer	Sc-15321	Rabbit polyclonal	IgG fraction	Human Homer-1b 13-354	10 ug/ml	1 ug/ml
Hsc70	Sc-7298	Mouse monoclonal	IgG(2a)	Human C-terminus peptide	10 ug/ml	1 ug/ml
Kv2.1	Ab5186	Rabbit polyclonal	IgG fraction	Rat C-terminus peptide	10 ug/ml	Not Done
mGluR1	G7794	Rabbit polyclonal	IgG fraction	Rat C-terminus peptide	10 ug/ml	Not Done
mGluR5	Ab27190	Rabbit polyclonal	Affinity purified	Human C-terminus peptide	10 ug/ml	1 ug/ml
Occludin	33-1500	Mouse monoclonal	IgG fraction	Human C-terminus 100 aa	10 ug/ml	1 ug/ml
NMDZ zeta 1	Sc-1467	Goat polyclonal	Affinity purified	Human C-terminus peptide	10 ug/ml	Not Done
PKC zeta	Sc-216	Rabbit polyclonal	Affinity purified	Rat C-terminus peptide	10 ug/ml	1 ug/ml
Piccolo	Sc-18569	Goat polyclonal	Affinity purified	Human C-terminus peptide	10 ug/ml	Not Done
Rabaptin-5	Sc-6162	Goat polyclonal	Affinity purified	Human C-terminus peptide	10 ug/ml	Not Done
Synaptotagmin VII	Sc-15420	Goat polyclonal	Affinity purified	Human C-terminus peptide	10 ug/ml	1 ug/ml
Stat2	Sc-476	Rabbit polyclonal	Affinity purified	Human C-terminus peptide	10 ug/ml	1 ug/ml
